# User Experience of a Bespoke Videoconferencing System for Web-Based Family Visitation for Patients in an Intensive Care Unit: 1-Year Cross-Sectional Survey of Nursing Staff

**DOI:** 10.2196/54560

**Published:** 2025-03-21

**Authors:** Aoife Murray, Irial Conroy, Frank Kirrane, Leonie Cullen, Hemendra Worlikar, Derek T O'Keeffe

**Affiliations:** 1 Health Innovation Via Engineering Laboratory, School of Medicine, University of Galway, University Road, Galway, H91TK33, Ireland, 353 091492147; 2 School of Medicine, College of Medicine Nursing and Health Sciences, University of GalwayGalway, Ireland; 3 Department of Medical Physics and Clinical Engineering, University Hospital Galway, Galway, Ireland; 4 Critical Care Department, University Hospital Galway, Galway, Ireland; 5 Lero Science Foundation Ireland Centre for Software Research, University of Galway, Galway, Ireland

**Keywords:** telemedicine, health, telehealth, videoconferencing, web-based, usability, intensive care, critical care, communication, COVID-19, SARS-COV-2, intensive care unit, ICU, cross-sectional survey, nursing, transmission, transmission risk, usability questionnaire, questionnaire, reliability, satisfaction, usefulness, family

## Abstract

**Background:**

During the COVID-19 pandemic, in-person visitation within hospitals was restricted and sometimes eliminated to reduce the risk of transmission of SARS-CoV-2. Many health care professionals created novel strategies that were deployed to maintain a patient-centered approach. Although pandemic-related restrictions have eased, these systems, including videoconferencing or web-based bedside visits, remain relevant for visitors who cannot be present due to other reasons (lack of access to transport, socioeconomic restraints, geographical distance, etc).

**Objective:**

The aims of this study were (1) to report the experience of intensive care nursing staff using a bespoke videoconferencing system called ICU FamilyLink; (2) to examine the scenarios in which the nursing staff used the system; and (3) to assess the future use of videoconferencing systems to enhance communication with families.

**Methods:**

A modified Telehealth Usability questionnaire was administered to the nursing staff (N=22) of an intensive care unit in a model 4 tertiary hospital in Ireland 1 year after implementing the bespoke videoconferencing system.

**Results:**

In total, 22 nurses working in the intensive care department at University Hospital Galway, Ireland, responded to the survey. A total of 23% (n=5) of participants were between the ages of 25 and 34 years, 54% (n=12) were between 35 and 44 years, and 23% (n=5) were between 45 and 54 years. Most (n=15, 68%) of the participants reported never using videoconferencing in the intensive care setting to communicate with family members before March 2020. The modified Telehealth Usability Questionnaire showed overall satisfaction scores for each subcategory of ease of use and learnability, interface quality, interaction quality, reliability, satisfaction and future use, and usefulness. In total, 21 (95%) participants agreed or strongly agreed with the statement, “I would use the ICU FamilyLink system in future circumstances in which family members cannot be physically present (ie, pandemics, abroad, inability to travel, etc),” and 1 participant responded neutrally. One participant highlighted a common scenario in intensive care settings in which a videoconferencing system can be used “Even without COVID, web-based communication is important when patients become unexpectedly ill and when families are abroad.”

**Conclusions:**

This study provides valuable insights into health care professionals’ experience using a videoconferencing system to facilitate web-based visits for families. We conclude that videoconferencing systems when appropriately tailored to the environment with the users in mind can be an acceptable solution to maintain communication with family members who cannot be physically present at the bedside. The bespoke videoconferencing system had an overall positive response from 22 nursing staff who interacted with the system at varying frequency levels.

## Introduction

### Background

In recent years, many intensive care departments have adopted a patient- and family-centered care approach. This includes having close family members physically present at the bedside during day-to-day activities and attending care meetings. Previous studies have highlighted the psychological and physical benefits of this approach for patients and their families during an individual’s inpatient stay in intensive care [1-3]. However, having family members physically present at the patient’s bedside can be difficult or even impossible due to geographical distance, financial challenges, and other family obligations. A significant barrier to bedside visits in the recent past was the COVID-19 pandemic.

The pandemic imposed severe and often complete restrictions on visits and forced health care professionals to change their traditional way of delivering patient care [4]. Prior to COVID-19, critical care nursing staff had been striving toward involving relatives in the intensive care unit (ICU) setting. However, many ICUs suspended these practices with the imposition of visitor restrictions [5]. These restrictive policies were introduced to decrease the risk of transmission of COVID-19 in health care settings [4]. These policies have been shown to be extremely isolating for patients, distressing for family members, and even hampering clinical care provided by health care professionals [1,6]. In particular, patients who are critically ill or vulnerable are often reliant on family for support, and at times, families are surrogate decision makers for patients. Health care professionals quickly realized the impact of visitor restrictions on family-centered care in intensive care settings. They called urgently for ways to maintain communication with family members who were no longer allowed to be physically present at the bedside [7]. At the beginning of the pandemic, hospitals quickly adopted technological solutions, including videoconferencing systems, to maintain or reopen communication channels. Previous studies have shown that adopting telemedicine solutions has numerous barriers, including the user’s acceptance and experience with the technology [8-10]. Other studies have shown positive patient experiences using videoconferencing systems for web-based family support during patient rounds. However, other studies identify challenges for health care professionals, including additional workload and difficulty learning and integrating new technology into clinical practice [11,12].

Prior to the pandemic, videoconferencing in the intensive care department was limited to tele-ICU and rarely used for remote family communication. At the start of the COVID-19 pandemic, ICUs worldwide adopted various modes of maintaining communication with family members who were not allowed to be physically present at the bedside due to the risk of transmission of COVID-19. Intensive care units often serve wide geographical areas, so videoconferencing systems have the potential to improve communication with patients’ families and reduce the psychological effects of intensive care admission on both patients and families. Videoconferencing systems can enable family members who cannot be physically present to be more involved during a patient’s intensive care journey.

### Aim of the Study

The aims of this study were (1) to report the experience of intensive care nursing staff using a bespoke videoconferencing system designed for patients and staff to communicate with patients’ families remotely during hospitalization in an intensive care department of a tertiary referral hospital that had no prior video-calling system in place; (2) to examine the scenarios that nursing staff used the videoconferencing system; and (3) to assess the future use of videoconferencing systems to enhance family communication in intensive care settings using a modified version of the validated Telehealth Usability Questionnaire (mTUQ) [13].

## Methods

### Overview of ICU FamilyLink

**Figure 1.**
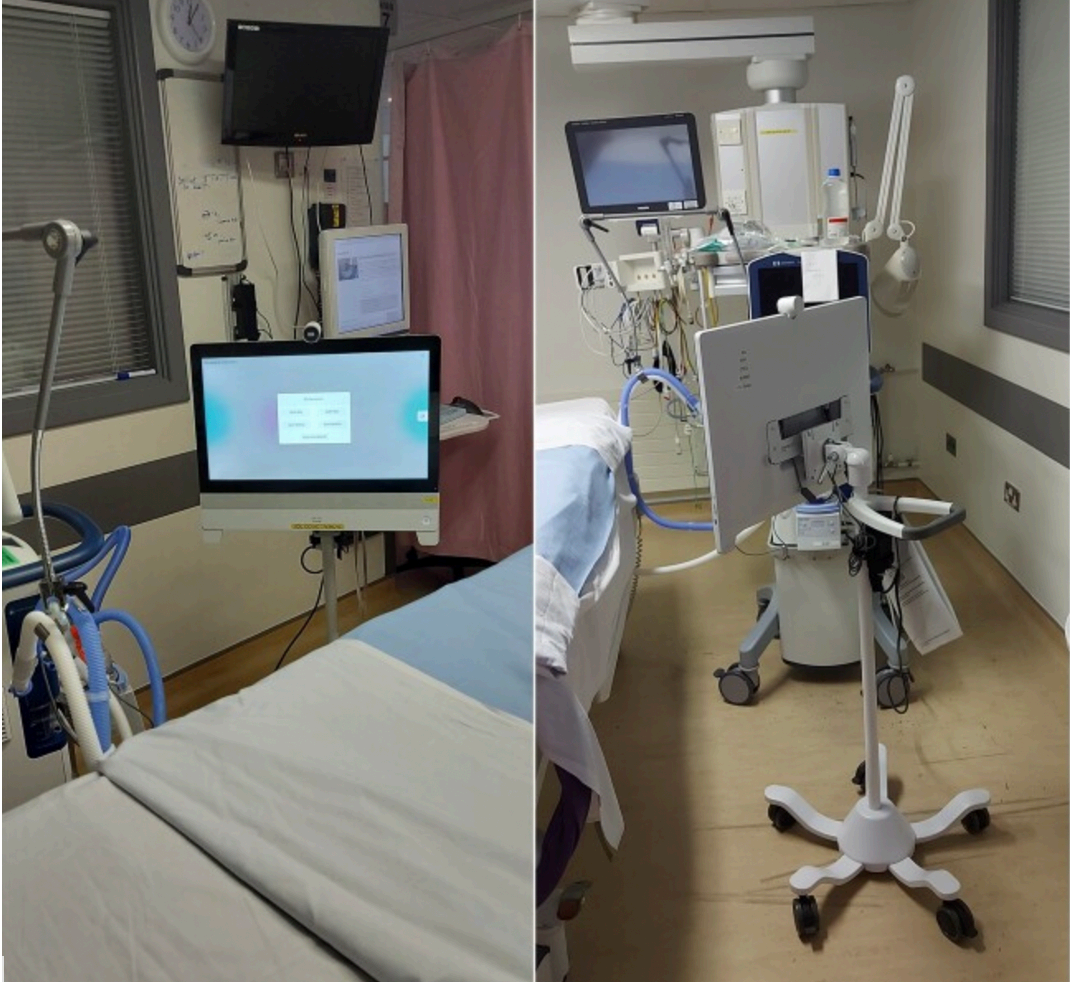
Video end point mounted on a mobile unit stand at an intensive care unit bedside.

A bespoke videoconferencing system called “ICU FamilyLink” was developed for the intensive care environment to allow for ad hoc web-based bedside visits available 24/7 with 1 or more close family members who may be in separate households [14]. The system was designed to be easy to use and to allow health care professionals to maintain appropriate control to maintain the security and privacy required for a hospital setting. The technology was chosen to deliver a reliable connection with high-quality audio and video. Several key requirements were previously identified during the iterative process of development [14]. This system used a 23-inch touch-screen video end point mounted on a mobile unit stand with a commercial cloud videoconferencing platform (Figure 1).

A simple menu was designed for ease of use and maximum video-call security. Functions such as recording, chat, and screen sharing were disabled for security and privacy. The home screen mirrored the department’s naming scheme for patient bed spaces and had the minimum number of clicks to connect. Three mobile video units were available for bedside web-based visits, and a stationary unit was available in a quiet room for medical staff to meet with families digitally.

A suite of documents, email templates, and multimedia was also created to support staff training, use, and troubleshooting. In addition, volunteer IT professionals operated a helpline for family members to ensure clinical staff were not burdened by technical queries outside their clinical training scope. A Data Protection Impact Assessment was completed, and the system was in keeping with hospital protocol and General Data Protection Regulation compliant.

The system was developed and rapidly introduced, with the first call within 2 weeks and the complete system rollout in under 3 weeks. Despite this rapid rollout, significant emphasis was placed on staff training, completion of relevant hospital risk assessments, and adherence to hospital policies. Focus on these areas was essential to ensure that staff, who were the main facilitators of the system, would find the system acceptable.

### Survey Development

The validated Telehealth Usability questionnaire (TUQ) was used with minor modifications to capture relevant information to the specific use case [13]. The modified questionnaire (mTUQ) was developed with input from clinicians, engineers, a medical physicist, and a senior intensive care nurse.

The survey consisted of 40 questions in total. It included 22 questions from the TUQ, using a 7-point Likert scale, to assess the ease of use and learnability, interface quality, interaction quality, reliability, satisfaction and future use, and usefulness. The remaining questions ascertained participants’ demographics and details on the respondent’s use of the videoconferencing system in the ICU. Free-text comment boxes were available to gather additional qualitative data.

### Recruitment

The mTUQ was distributed in paper form and via a web-based survey 1 year after the ICU FamilyLink was deployed. This allowed the system to become established in the department and minimized any bias that may have been created at the peak of the pandemic. All ICU nursing staff were eligible to participate in this study. Nursing staff were the primary users and facilitators of the ICU FamilyLink system. The system was mainly used for web-based bedside visits, rather than formal medical updates from the ICU medical team, although it was available if needed. The survey was open to any ICU staff member. Staff were recruited via emails, verbal reminders at nursing handover, and paper copies of surveys left in break rooms and main staff areas.

### Data Analysis

Data were exported from the web-based survey and manually inputted from the paper surveys into a Microsoft Excel spreadsheet for analysis.

### Ethical Considerations

Ethics approval for this study was obtained from the Galway Hospital Clinical Research Ethics Committee (ref C.A. 2674), and written informed consent, including web-based signatures for web-based participants was obtained from all respondents. All respondents participated voluntarily, could opt out at any point, and did not receive financial compensation. All data were anonymized.

## Results

A total of 22 nurses who had been working in the intensive care unit at University Hospital Galway responded to the survey. All the respondents had used the ICU FamilyLink system at least once, although prior usage was not a requirement for participation in this study.

### Characteristics of Participants

All participants were nursing staff, of these, 18% (n=4) were nurse managers. In total, 23% (n=5) of participants were between the ages of 25 and 34 years, 54% (n=12) were between 35 and 44, and 23% (n=5) were between 45 and 54 years. A total of 68% (n=15) of participants reported that they had never used videoconferencing in the intensive care setting to communicate with family members prior to March 2020 (Table 1).

During the initial introduction of the system, short training sessions were held, “superusers” were trained, and an instruction manual and video training were created. The survey showed that 36% (n=8) of participants watched a colleague before independent use and 77% (n=17) asked a colleague to teach them.

**Table 1.** Baseline characteristics of respondents.

	Respondents (N=22), n (%)
**Age categories (years)**	
25-34	5 (23)
35-44	12 (54)
45-55	5 (23)
**Role**	
Staff nurse	18 (82)
Nurse manager	4 (18)
**Prior experience with any videoconferencing software in the critical care setting to communicate with patient’s family members**	
Yes	7 (32)
No	15 (68)
**Frequency of use (number of video calls)**	
1-2	6 (27)
3-5	3 (14)
6-10	4 (18)
10-20	4 (18)
>20	5 (23)

### Participants’ Use of ICU FamilyLink

The system’s usage was examined based on the frequency of video calls made (Table 1) and various scenarios (Table 2). In total, 27% (n=6) of participants used the system only 1-2 times, while 23% (n=5) of participants used the system over 20 times. Participants most commonly used the system for family time with patients while the staff were present (n=19, 86%), family time with the patient without clinical staff present (n=15, 68%), and providing daily progress updates (n=10, 45%). In total, 27% (n=6) used the system for remote family support while 1 family member was physically present at the bedside. While 9% (n=2) of respondents used the system to deliver bad news, 14% (n=3) used the system for an end-of-life scenario; 1 respondent reported using the system for a formal care team meeting with the family. In addition, respondents were asked what purposes they felt the system was not suitable for (Table 3). A total of 45% (n=10) of respondents felt that delivering bad news and end of life were not suitable scenarios for using the videoconferencing system (Table 3). One respondent commented that, “Ideally, you would give bad news in person, but it could be used if not possible.”

**Table 2.** Use of the ICU FamilyLink system.

Scenarios that the respondent has used the ICU FamilyLink system	Respondents (N=22), n (%)
Family time with the patient (while clinical staff carry out routine care)	19 (86)
Family time with the patient (without clinical staff present)	15 (68)
Daily progress updates	10 (45)
Remote family support for a designated in-person family member	6 (27)
End of life	3 (14)
Giving bad news	2 (9)
Formal care team meeting or family meeting	1 (4)
Education session for family	0 (0)

**Table 3.** Scenarios that participants felt were not suitable for the ICU FamilyLink system.

Clinical scenarios	Respondents (N=22), n (%)
Giving bad news	10 (45)
End of life	10 (45)
All are suitable	7 (32)
Formal care team meeting or family meeting	3 (14)
Education session for family	2 (9)
Daily progress updates	1 (4)
Other	1 (4)
Family time with the patient (without clinical staff present)	1 (4)
Family time with the patient (while clinical staff carry out routine care)	1 (4)
Remote family support for a designated in-person family member	0 (0)

### Usability Attributes of the Videoconferencing System Using the mTUQ

The mTUQ showed an overall positive satisfaction score for all subcategories, including ease of use and learnability, interface quality, interaction quality, reliability, satisfaction and future use, and usefulness (Table 4).

**Table 4.** Responses to the modified Telehealth Usability questionnaire (N=22).

	Strongly disagree, n (%)	Disagree, n (%)	Somewhat disagree, n (%)	Neutral, n (%)	Somewhat agree, n (%)	Agree, n (%)	Strongly agree, n (%)
**Ease of use and learnability**							
1. It was easy to learn how to set up and use the system.	0 (0)	0 (0)	2 (9)	3 (14)	6 (27)	3 (14)	8 (36)
2. It was simple to use the system.	0 (0)	0 (0)	3 (14)	2 (9)	8 (36)	2 (9)	7 (32)
3. Compared to facilitating and managing in-person visits, the FamilyLink system was a more time-efficient way to engage with families.	0 (0)	2 (9)	1 (4)	4 (18)	7 (32)	6 (27)	2 (9)
**Interface quality**							
4. I am able to navigate setup, initiate, and complete calls without difficulty.	0 (0)	1 (4)	2 (9)	4 (18)	4 (18)	3 (14)	8 (36)
5. The onscreen menu for FamilyLink was intuitive to navigate.	0 (0)	1 (4)	0 (0)	2 (9)	5 (23)	6 (27)	8 (36)
6. The ICU FamilyLink system could do everything I wanted it to do.	0 (0)	0 (0)	1 (4)	2 (9)	4 (18)	5 (23)	10 (45)
**Interaction quality**							
7. The video quality was good and provided a clear 2-way conversation between me (and the patient, when able) and the family members.	0 (0)	1 (4)	0 (0)	0 (0)	3 (14)	4 (18)	14 (64)
8. The audio quality was good and provided clear 2-way conversation between me (and the patient, if able) and their family members.	0 (0)	0 (0)	1 (4)	1 (4)	1 (4)	5 (23)	14 (64)
9. I prefer the ICU FamilyLink monitor on a stand rather than a handheld device or smaller tablet.	0 (0)	0 (0)	0 (0)	6 (27)	1 (4)	5 (23)	10 (45)
10. I was able to express myself effectively using the FamilyLink system.	0 (0)	0 (0)	0 (0)	1 (4)	6 (27)	5 (23)	10 (45)
11. Compared to telephone conversations, the ICU FamilyLink was better to communicate and expressing important messages.	0 (0)	1 (4)	2 (9)	2 (9)	3 (14)	6 (27)	8 (36)
**Reliability**							
12. The system was reliable and consistently facilitated video calls.	0 (0)	0 (0)	1 (4)	2 (9)	4 (18)	4 (18)	11 (50)
13. I feel the FamilyLink system facilitates private and secure communication.	2 (9)	2 (9)	0 (0)	3 (14)	3 (14)	8 (36)	4 (18)
14. Whenever I made a mistake using the ICU FamilyLink system, I could quickly recover.	0 (0)	0 (0)	0 (0)	4 (18)	4 (18)	10 (45)	4 (18)
15. Family members were able to follow the emailed instructions and connect to a web-based visit call without additional technical assistance from me or other health care staff.	0 (0)	1 (4)	0 (0)	2 (9)	7 (32)	6 (27)	6 (27)
**Satisfaction and future use**							
16. I feel comfortable communicating with my patient’s family members using the FamilyLink system.	0 (0)	0 (0)	0 (0)	1 (4)	5 (23)	8 (36)	8 (36)
17. The ICU FamilyLink system is an acceptable way to communicate while visitors are restricted.	0 (0)	0 (0)	0 (0)	0 (0)	2 (9)	9 (41)	11 (50)
18. I would use the ICU FamilyLink system in future circumstances in which family members cannot be physically present (ie, pandemics abroad and inability to travel).	0 (0)	0 (0)	0 (0)	1 (4)	0 (0)	6 (27)	15 (68)
19. I will use the ICU FamilyLink system in addition to in-person visits in the future.	0 (0)	0 (0)	1 (4)	2 (9)	2 (9)	7 (32)	10 (45)
20. I would recommend the ICU FamilyLink system in other health care settings.	0 (0)	0 (0)	0 (0)	1 (4)	1 (4)	4 (18)	16 (73)
**Usefulness**							
21. The ICU FamilyLink system provides for continuity of communication while visiting is limited.	0 (0)	0 (0)	0 (0)	0 (0)	2 (9)	5 (23)	15 (68)
22. The ICU FamilyLink system has positively impacted the overall care of patients in critical care during the visitor restrictions.	0 (0)	0 (0)	0 (0)	1 (4)	3 (14)	5 (23)	13 (59)

### Ease of Use and Learnability

The majority of nursing staff responded positively to ease of use and learnability questions. Interestingly, when specifically asked to compare the time efficiency of organizing in-person visits versus web-based visits (question 3), 18% (n=4) of participants responded neutrally, 14% (n=3) provided negative responses, and 68% (n=15) gave positive responses (Table 4, questions 1-3).

Two comments were made about the process of sending the videoconferencing link to family members, indicating the need to improve the process.

*The equipment itself was very easy to use, it can be a pain to log out of the computer and relog in, an app on the computer at the nurses station would be better.*

[Respondent who used the system 10-20 times]

*I think it would be beneficial if the email sent to the families to download the software could be sent to every computer in the unit.*

[Respondent who used the system more than 20 times]

These comments indicate an area for improvement to reduce the burden of work for staff members and highlight the frustration staff commonly feel when newly introduced technology adds extra tasks. There are limitations to fully integrating technology; however, decreasing the administration time for the clinical staff could lead to better technology uptake. Despite this feedback, the 2 participants reported frequent use of the system.

### Interface Quality

Nursing staff most frequently responded with “strongly agree” to the interface quality questions, question 4 (n=8; 36%), question 5 (n=8; 36%), and question 6 (n=10; 45%), respectively (Table 4).

Free text comments from 2 participants included “very simple and easy to use” and “good quality.”

### Interaction Quality

A total of 64% (n=14) participants strongly agreed that the video and audio quality was good and provided clear 2-way conversation. When asked about their preference for the ICU FamilyLink monitor on a stand rather than a handheld device or smaller tablet, the majority of participants (n=16, 73%) responded in the positive, only 6 (27%) participants were neutral, and none responded in the negative. Most of the respondents (n=17, 77%) felt that compared to telephone conversations, the ICU FamilyLink was better for communicating and expressing important messages (Table 4, questions 7-11).

One participant highlighted the importance of network coverage for family members, though beyond the hospital’s control, was an important factor: “Very dependent on internet coverage for the family.” Another participant indicated that the visual aspect of the system was better than phone communication: “It helped build a connection with families that isn’t possible via phone.”

### Reliability

Overall, respondents more frequently responded positively to reliability (Table 4, questions 12-15). However, 4 (18%) respondents disagreed or strongly disagreed with the statement, “I felt the ICU FamilyLink system facilitates private and secure communication.”

### Satisfaction and Future Use

All participants responded positively to the statement, “The ICU FamilyLink system is an acceptable way to communicate while visitors are restricted.” In total, 21 (95%) participants agreed or strongly agreed with the statement, “I would use the ICU FamilyLink system in the future circumstances in which family members cannot be physically present (i.e., pandemics, abroad, inability to travel, etc),” and 1 participant responded neutrally (Table 4, questions 16-20).

Qualitative data also indicate health care professionals’ positive feedback and wish to use the system in the future:

 *Even without COVID, online communication is important when patients become unexpectedly ill, and families are abroad.*

 *Extremely valued by family members, I have had limited exposure to its use due to working mostly night shifts.*

*Familylink has been a lifesaver during the pandemic; in my opinion, it is very easy to use and great for patients to be able to see their relatives onscreen while visiting is restricted. I will use it at any opportunity.*

### Usefulness

In total, 21 (95%) participants responded positively to questions about usefulness (Table 4, questions 21 and 22), except 1 neutral response to question 22: “The ICU FamilyLink system has positively impacted the overall care of patients in intensive care during the visitor restrictions.”

## Discussion

### Overview

With an overall shift toward a patient- and family-centered approach, health care professionals can harness technology to enable and optimize this approach. However, with the introduction of any technology into a new environment, particularly one as complex as health care delivery, careful design, assessment, and research should be completed to ensure acceptability, usability, and impact on all users. While health care professionals are trained to a high degree in health and medicine, their training, understanding, and use of technology vary widely.

### Strengths, Limitations, and Future Directions

This study included participants who had only interacted with the ICU FamilyLink system a handful of times and also very frequent users. It also was inclusive for all age ranges and had both staff grade and senior nurses. In usability studies, it is vital to capture input from all spectrums of technical users.

One limitation of this study was that it did not interview or survey patients or their families. While this was considered, due to limited staff and stressful circumstances for patients and families, it was not included as a component of the study. There have been studies recently published examining family and patient experiences in using both phone and video for web-based visits [15-17]. Staff acceptance is critical for implementing systems like the ICU FamilyLink into regular practice. ICU staff are usually required to facilitate these calls due to the dependency needs of the ICU patients.

This study, like many similar studies, was conducted under pandemic conditions. The TUQ was modified to account for these conditions. It is encouraging that responses to questions on usefulness and future use are overall positive, with 68% (n=15) of respondents “strongly agreeing” that they would use the ICU FamilyLink system in future circumstances in which family members cannot be physically present (question 18). While there are positive indications for postpandemic use and its potential value is noted by the staff members, confirming these results outside of pandemic conditions would be important.

### Conclusions

The findings of this study provide valuable insights into health care professionals’ experiences using a videoconferencing system to facilitate web-based bedside visits for family members. We conclude that when appropriately tailored to the environment and with the users in mind, videoconferencing systems can be an acceptable solution for maintaining communication with family members who cannot be physically present at the bedside. Further studies are needed to better understand usability factors for technology in order to enhance and augment communication with ICU patients, staff, and patients’ family members.

## Abbreviations

ICU: intensive care unit
mTUQ: modified Telehealth Usability Questionnaire
TUQ: Telehealth Usability Questionnaire
